# Distinct sociodemographic differences in incidence and survival rates for human papillomavirus (HPV)-like, non-HPV-like, and “other”-like oral cavity and pharynx cancers: An analysis of Surveillance, Epidemiology and End Results (SEER) Program data

**DOI:** 10.3389/fonc.2022.980900

**Published:** 2022-08-18

**Authors:** Kelsey H. Jordan, James L. Fisher, Electra D. Paskett

**Affiliations:** ^1^ Division of Population Sciences, Comprehensive Cancer Center, The Ohio State University, Columbus, OH, United States; ^2^ Division of Epidemiology, College of Public Health, The Ohio State University, Columbus, OH, United States; ^3^ Arthur G. James Cancer Hospital and Richard J. Solove Research Institute, Columbus, OH, United States; ^4^ Division of Cancer Prevention and Control, Department of Internal Medicine, College of Medicine, The Ohio State University, Columbus, OH, United States

**Keywords:** human papillomavirus (HPV), tobacco, SEER program, risk factors, head and neck neoplasms, mouth neoplasms, pharyngeal neoplasms

## Abstract

**Purpose:**

Oral cavity and pharynx cancer (OCPC) cases are traditionally dichotomized into human papillomavirus (HPV) and non-HPV types. Using a proxy for HPV status, the objective was to evaluate differences in incidence and survival rates of OCPC anatomic sub-sites identified as: 1) HPV-like; 2) non-HPV-like (i.e., tobacco/alcohol-related); and 3) “other”-like (i.e., not predominantly HPV-like nor tobacco/alcohol-like) OCPCs.

**Methods:**

Data from the Surveillance, Epidemiology and End Results Program were used to examine incidence and survival rates for OCPC categories over time and according to age, sex, race, ethnicity, stage at diagnosis, neighborhood socioeconomic status (i.e., nSES or Yost Index), and rurality/urbanity (i.e., Rural Urban Commuting Area (RUCA) codes). Although HPV status was unavailable in this dataset, OCPC anatomies and histologies were classified into three sub-categories, based on potential risk factors. Frequencies, average annual, age-adjusted incidence rates, five-year relative survival rates, and 95% confidence intervals were examined across and within OCPC categories.

**Results:**

HPV-like OCPC incidence rates sharply increased from 1975 through 2015 while non-HPV-like and “other”-like OCPC rates decreased, all converging to similar rates from 2016 through 2018. Increasing over time for both categories, survival was highest for HPV-like and lowest for non-HPV-like OCPCs; survival for “other”-like OCPCs remained stable. Generally, across OCPC categories, incidence and survival rates were significantly higher among males vs. females, Whites vs. African Americans, and non-Hispanics vs. Hispanics. “Other”-like OCPC incidence decreased with increasing nSES tertiles, while no nSES differences were observed for HPV-like and non-HPV-like OCPCs. Incidence rates were significantly lower among urban (vs. rural) residents. For all OCPC categories, survival rates were significantly higher with increasing nSES and variable across RUCA categories.

**Conclusion/Impact:**

HPV-like and non-HPV-like OCPC cases had distinct sociodemographic differences; “other”-like OCPC cases were a sociodemographic blend of HPV-like and non-HPV-like OCPC cases, resembling more of the sociodemographic makeup of non-HPV-like OCPC cases. To prevent new OCPCs, additional studies are needed to epidemiologically and clinically differentiate between OCPC categories so that high-risk groups can be better targeted in future public health interventions.

## Introduction

In 2022, it was estimated that 54,000 incident cases of oral cavity and pharynx cancer (OCPC) would be diagnosed in the United States (US) and 11,230 people would die from the disease (1). The incidence rate has increased by 0.8% per year from 2009 to 2019 (1). The 2015-2019 average annual, age-adjusted OCPC incidence rate in the US was 11.5 per 100,000 persons; the mortality rate was 2.5 per 100,000 persons (2). The five-year relative survival probability (hereafter, referred to as survival rate) for those diagnosed with OCPC is 68.0% (2).

OCPC incidence, mortality, and survival rates vary across anatomical sites and demographics. Anatomically, OCPCs span from the nasopharynx through the hypopharynx, including specific anatomies of the oral cavity and oropharyngeal region (3). Like causal pathways for many diseases, those for OCPC are complex and multi-factorial, with causes interacting with one another. The aetiology is typically described using the primary, required, and/or common risk factor with cases being labeled by their most common risk factor (4–6).

The human papillomavirus (HPV) and tobacco and alcohol use (hereafter, referred to as tobacco/alcohol use) have consistently been identified as common OCPC risk factors (3, 7–10). Tobacco/alcohol use can be a risk factor for HPV and vice versa. Therefore, the risk factors of tobacco/alcohol use and HPV can act independently, complementarily, or synergistically in OCPC cases (11, 12). Studies have shown that when both HPV and tobacco/alcohol use are present in OCPCs that HPV is the predominant risk factor, making tobacco/alcohol use an underlying risk factor and identifying such OCPC cases as HPV-related (5, 8, 13); when HPV is not present and tobacco/alcohol use is present, such OCPC cases are identified as tobacco/alcohol-related (9). There may be instances where other risk factor(s) (i.e., neither HPV nor tobacco/alcohol use) are identified as primary in the multi-factorial causality of OCPC, creating 3+ categories of OCPCs. Confirmed by HPV-testing of specific OCPC sub-anatomies (13), these disparate biological risk factors have been implicated for specific anatomies of OCPCs (3, 8, 14–16).

As confirmed by HPV testing, HPV has been associated with more than 70% of all US cancers found in the oropharyngeal region of the head and neck (17), namely the tonsils and base of the tongue (8, 13, 14, 16, 18, 19). HPV-related OCPC diagnoses have increased by 46.3% from 1973 to 2018 (2, 16, 19–21). Occurring most often in males who are infrequent smokers or non-smokers (22), HPV-related OCPC mortality rates are also significantly increasing at a similar pace (16). Interestingly, more affluent and educated persons seem to be disproportionately affected by HPV-related OCPCs (22), possibly explaining the higher survival rate for those diagnosed with HPV-related OCPCs, compared with those diagnosed with non-HPV-related OCPCs (22).

Tobacco use and alcohol consumption are primary non-HPV-related OCPC risk factors (9). These OCPCs predominantly occur in the oral cavity, especially the front of the mouth (e.g., lips, buccal mucosa, gingiva) where tobacco/alcohol substances most frequently contact the oral cavity (3, 14, 16). Non-HPV-related OCPCs were the most prevalent OCPCs until the early 2000s (14), but incidence and mortality rates have decreased over time, significantly so for lip cancer among males and floor of mouth cancer among males and females (16); this is likely attributable to decreased tobacco use in recent years (23). Lower socioeconomic status (SES) groups are still disproportionately affected by non-HPV-related OCPCs and have lower survival rates than groups with higher SES (22).

Additional specific anatomies of OCPCs likely remain un(der)investigated. The OCPC region is expansive and only cancers from very well-defined specific anatomies have been routinely HPV-tested and associated with, and categorized by, known risk factors (e.g., HPV, tobacco/alcohol use). Cancers among these additional specific anatomies are likely indicative of other OCPC risk factor(s) and therefore are not predominantly associated with HPV positivity nor tobacco/alcohol use. For instance, HPV-related and non-HPV-related (e.g., alcohol- and tobacco-related) OCPCs are less frequently diagnosed in the hypopharynx (18, 24).

There is limited recent epidemiological information on OCPC anatomic sites that are well-established as associated with HPV, tobacco/alcohol use, and/or unidentified risk factors. This is especially concerning given the rising incidence rates of some OCPC specific anatomies, including (lingual, palatine) tonsils and tongue (8, 16, 25). Further, epidemiological differences based on sociodemographics, including neighborhood SES (nSES) (26, 27) and rurality/urbanity (20, 28), have been suggested, but remain minimally investigated; these measures are important because they may help identify populations at higher risk and in need of prevention efforts. Therefore, the objective of this paper was to evaluate epidemiological differences in incidence and survival rates, including those based on nSES and rurality/urbanity, among other potential demographic and clinical risk factors, for three aetiologically based OCPC categories termed: 1) HPV-like, 2) non-HPV-like, and 3) “other”-like OCPCs using a large, national, well-annotated database.

## Materials and methods

### SEER Program databases

The Surveillance, Epidemiology and End Results (SEER) Program is a collection of population-based central cancer registries capturing facts from 22 geographic areas representing 48.0% of the US population (29). The following four SEER datasets comprised of different SEER registries were used to characterize: 1) recent incidence rates and frequency distributions according to demographic and clinical information (30); 2) incidence trends (31); 3) survival rates according to demographic and clinical information (32); and 4) recent incidence rates, frequency distributions, and survival rates according to nSES and rurality/urbanity, using a specialized dataset with permission from the SEER Program (33). Institutional review board approval was not necessary and ethical consent was not required because SEER data are publicly available and deidentified.

### OCPC risk factor categorization

Chaturvedi et al.’s specific anatomic and histologic methodology (14) (based on International Classification of Disease for Oncology version-3, ICD-O-3, topography codes) distinguishes between “potentially HPV-related and HPV-unrelated” OCPCs when HPV status is unknown. Prior HPV testing of a national sample of OCPCs also supports Chaturvedi et al.’s classifications (13). Moreover, as consistently demonstrated in many molecular and epidemiologic studies, HPV+ (or related) oral cancers have predominantly developed within the oropharyngeal region, especially the base of the tongue and lingual and palatine tonsils (8).

Here, cancers listed within the OCPC category of the SEER variable ‘Site Recode’ were investigated. Given that the SEER datasets used in these analyses did not include HPV status for the OCPC diagnoses, Chaturvedi et al.’s methodology (14) was utilized to classify OCPCs of specific anatomies into aetiological risk factor sub-categories. The SEER variable ‘Primary Site’ was used to idenitfy specific anatomies for each of the three OCPC aetiological risk factor sub-categories of HPV-like (i.e., Chaturvedi et al.’s “potentially HPV-related”), non-HPV-like (i.e., Chaturvedi et al.’s “potentially HPV-unrelated” (e.g., alcohol- and tobacco-related)), and “other”-like (i.e., Chaturvedi et al.’s excluded anatomies and/or histologies) OCPCs.

HPV-like OCPC sites included the specific anatomies of base of tongue (ICD-O-3: C019), (palatine, lingual) tonsil (ICD-O-3: C024, C090-099), and oropharynx (ICD-O-3: C100-109). Non-HPV-like OCPCs included the specific anatomies of the tongue (non-base; ICD-O-3: C020-023, C025-029), gum (ICD-O-3: C030-039), floor of mouth (ICD-O-3:C040-049), hard and soft palates (ICD-O-3: C050-059), and other/unknown sub-anatomies of the mouth (ICD-O-3: C060-069). HPV-like and non-HPV-like OCPCs were further restricted to specific histologies (i.e., squamous cells) using the variable ‘ICD-O-3 Histology/Behavior’ (ICD-O-3: 8050-8076, 8078, 8083, 8084, 8094). “Other”-like OCPCs included those not included in the HPV-like and non-HPV-like categories, either because they were not one of the specific anatomies (i.e., lip (ICD-O-3: C000-009), nasopharynx (ICD-O-3: C110-119), salivary gland (ICD-O-3: C080-089), hypopharynx (ICD-O-3: C130-193), and other uncategorized OCPCs (ICD-O-3: C140-148)) nor histologies (i.e., non-squamous cells) described as being predominantly associated with either HPV or tobacco/alcohol use (13, 14).

### Sociodemographic factors

Incidence rates, frequency distributions, and survival rates were examined according to sex, age at diagnosis, race, ethnicity, and stage at diagnosis. Sex was characterized as ‘Male’ or ‘Female’ and age at diagnosis was described using five-year age groups. Race was characterized using the variable ‘Race Recode’, which categorized race as ‘White’, ‘Black’, ‘Asian or Pacific Islander’, ‘American Indian or Alaskan Native’, or ‘unknown’. Ethnicity was categorized as ‘Spanish-Hispanic-Latino’ (i.e., Spanish or Hispanic or Latino) or ‘non-Spanish-Hispanic-Latino’ (i.e., not Spanish nor Hispanic nor Latino), using the variable ‘Origin Recode NHIA’. Stage at diagnosis was characterized using ‘SEER Combined Summary Stage’ and was categorized as ‘*in situ*’, ‘Localized’, ‘Regional’, ‘Distant’, and ‘Unknown/unstaged’. Invasive cancers were of primary interest; *in situ* cancers were only included when stage at diagnosis was considered.

The nSES, or Yost, Index in SEER is a time-dependent composite score, including census tract-level information about education index, household income, percent below 150% of poverty line, median house value, percent unemployed, median rent, and percent working class variables (34). nSES scores have been categorized into tertiles, with the first and third tertiles representing lower and higher nSES, respectively. Census tract-level rurality data included in SEER are based on the US Department of Agriculture’s Rural Urban Commuting Area (RUCA) codes (35), with four categories (‘All Urban’, ‘Mostly Urban’, ‘Mostly Rural, ‘All Rural’).

### Statistical analyses

Recent (2014-2018) average annual, age-adjusted incidence rates, per 100,000 persons, frequency distributions, and 95% confidence intervals (CI) were calculated using SEER*Stat software (version 8.3.9). Age-adjustment of incidence rates were calculated using the 2000 U.S. standard population (36). For examination of incidence rates according to nSES and RUCA categories, cases diagnosed from 2012 through 2016, were included, as these represented the most recent years available. Incidence rate CI calculations were based on a method presented by Tiwari et al. (37).

Survival rates and associated 95% CIs were also calculated using SEER*Stat software for cases diagnosed from 2011 through 2017, with follow-up through 2018. For survival rates according to nSES and RUCA categories, cases diagnosed from 2009 through 2015, with follow-up through 2016, were used, as these represented the most recent years available. Survival rates are based on relative survival, a net survival measure representing cancer survival in the absence of other causes of death; it is the ratio of the proportion of observed survivors in a cohort of cancer patients to the proportion of expected survivors in a comparable set of cancer-free individuals.

For both incidence and survival rates, non-overlapping 95% CI’s were used to indicate statistically significant differences between levels of sociodemographic factors. Additionally, late-stage only cancers were examined for variations in incidence and survival rates according to nSES and RUCA categories.

## Results

**Figure 1:**
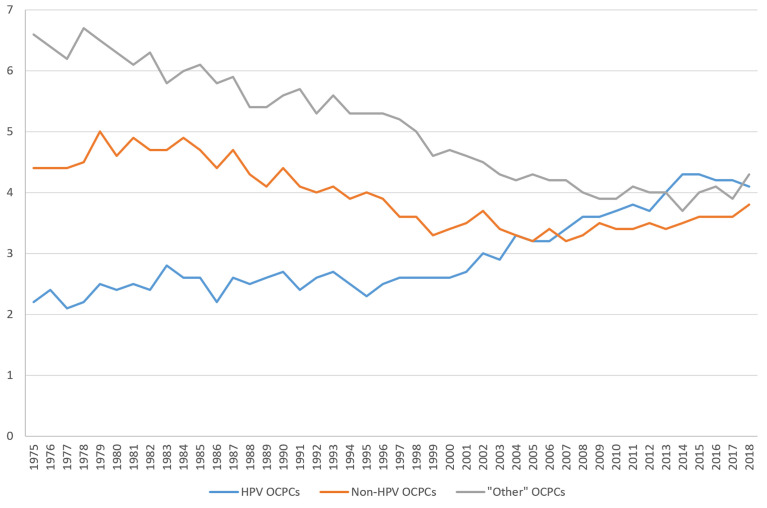
Trends in United States incidence rates (per 100,000) of oral cavity and pharynx cancers from 1975 to 2018 according to groupings of human papillomavirus (HPV)-like association*. *Surveillance, Epidemiology, and End Results (SEER) Program (www.seer.cancer.gov) SEER*Stat Database: Incidence - SEER Research Data, 9 Registries, Nov 2020 Sub (1975-2018) - Linked To County Attributes - Time Dependent (1990-2018) Income/Rurality, 1969-2019 Counties, National Cancer Institute, DCCPS, Surveillance Research Program, released April 2021, based on the November 2020 submission.

OCPC incidence rates have shifted over time (**Figure 1**). From 1975 to the early 2000s, incidence rates were highest for “other”-like OCPCs followed by non-HPV-like OCPCs. Since approximately 2001, incidence rates for HPV-like OCPCs have sharply increased, surpassing non-HPV-like and “other”-like OCPC rates, respectively. Incidence rates for all three OCPC categories have begun to converge from 2016 to 2018 (**Figure 1**).

**Figure 2:**
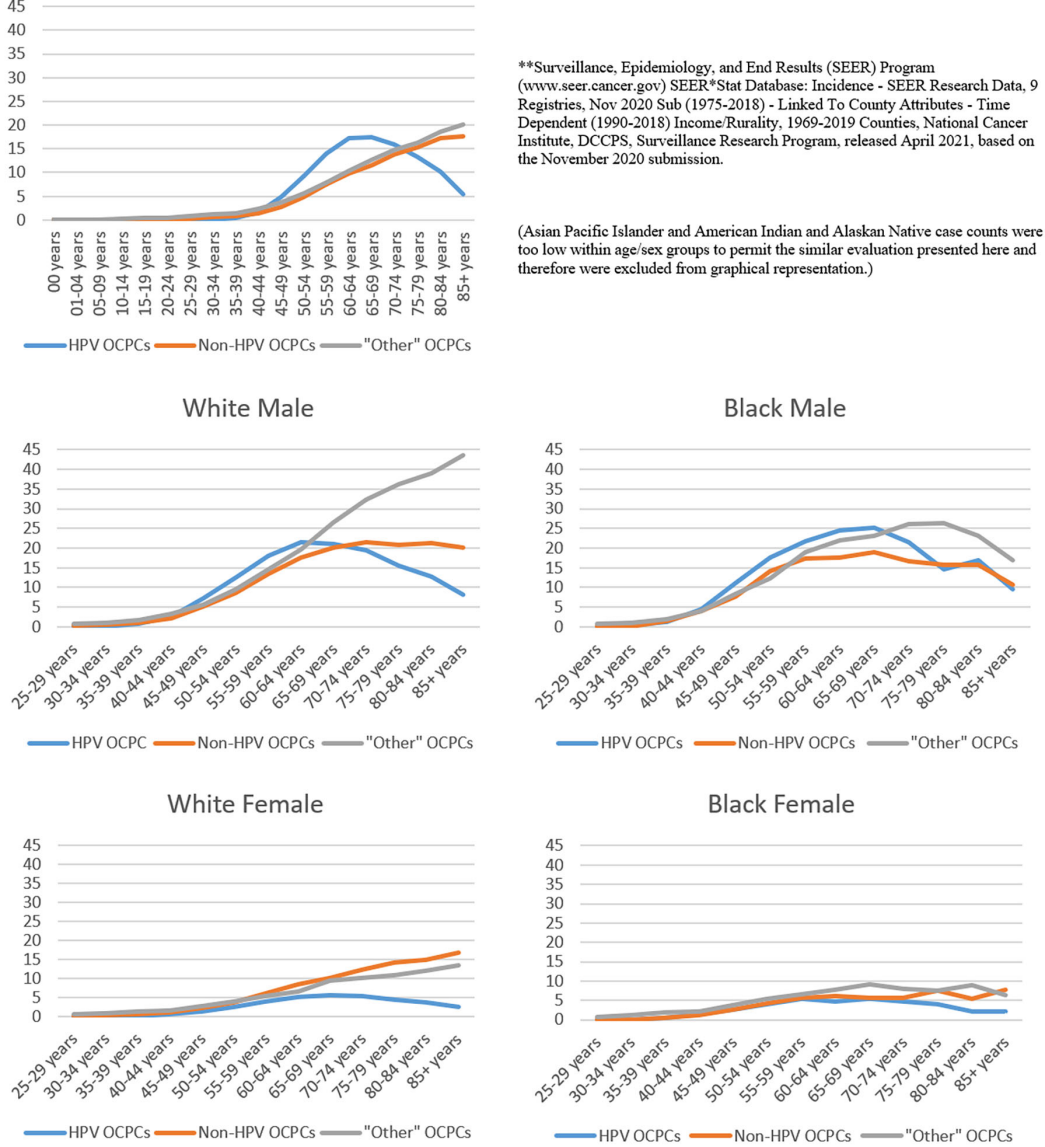
Age-specific, average annual (2014-2018) incidence rates (per 100,000) of oral cavity and pharynx cancers for various sex/race groups according to groupings of human papillomavirus (HPV)-like association**. **Surveillance, Epidemiology, and End Results (SEER) Program (www.seer.cancer.gov) SEER*Stat Database: Incidence - SEER Research Data, 9 Registries, Nov 2020 Sub (1975-2018) - Linked To County Attributes - Time Dependent (1990-2018) Income/Rurality, 1969-2019 Counties, National Cancer Institute, DCCPS, Surveillance Research Program, released April 2021, based on the November 2020 submission. (Asian Pacific Islander and American Indian and Alaskan Native case counts were too low within age/sex groups to permit the similar evaluation presented here and therefore were excluded from graphical representation.).

**Figure 2** shows age-specific, average annual incidence rates, overall and for specific sex/race groups. For all sex/race groups combined, the HPV-like OCPC incidence rate peaked at ages 65-69 years, while non-HPV-like and “other”-like OCPC incidence rates continued to increase with advancing age. Sex/race-specific OCPC incidence rates tended to peak in or near the age groups of 60-69 years, regardless of OCPC type, occurring at younger ages in males than females.

There were 65,538 invasive OCPCs diagnosed from 2014 through 2018; of these, 39.9% were HPV-like, 29.2% were non-HPV-like, and 30.9% were “other”-like OCPCs (**Table 1**). Incidence rates, regardless of categorization, were significantly higher among males and non-Hispanics. For HPV-like and non-HPV-like OCPCs, incidence rates were significantly higher among Whites; for “other”-like OCPCs, the incidence rate was significantly higher among Asian or Pacific Islanders (**Table 1**). The incidence rates were significantly higher for regionally staged HPV-like OCPCs and locally staged non-HPV-like and “other”-like OCPCs, compared to those diagnosed at additional stages (**Table 1**). HPV-like and non-HPV-like OCPCs occurred at similar rates across nSES tertiles; whereas, incidence rates for “other”-like OCPCs decreased with increasing nSES tertile. For each OCPC category, incidence rates were significantly lower among residents of urban census tracts; non-urban residents with HPV-like OCPCs had the highest incidence rates (**Table 1**).

**Table 1:** Average annual (2014-2018), age-adjusted incidence rates (per 100,000) of oral cavity and pharynx cancer (OCPC) cases by demographic and census tract-based neighborhood socioeconomic status (nSES, Yost Index) and Rural-Urban Commuting Area (RUCA) according to groupings of human papillomavirus (HPV)-like association.

	HPV-like OCPCs[95% CI]*; (frequency) n=26,154		Non-HPV-like OCPCs[95% CI]*; (frequency) n=19,112		“Other”-like OCPCs[95% CI]*; (frequency) n=20,272
Sex^1a^					
Male Female	**7.4 [7.3, 7.5]**; (83.1%) 1.3 [1.3, 1.4]; (16.9%)		**4.2 [4.2, 4.3]**; (58.6%) 2.6 [2.5, 2.6]; (41.4%)		**5.7 [5.6, 5.7]**; (66.0%) 2.5 [2.5, 2.6]; (34.0%)
Race^1a^					
White African American American Indian/Alaska Native Asian or Pacific Islander Unknown**	**4.7 [4.7, 4.8]**; (87.8%) 3.1 [3.0, 3.2]; (8.2%) 2.3 [2.0, 2.7]; (0.6%) 1.2 [1.1, 1.2]; (2.6%) — [—, —]; (0.8%)		**3.6 [3.5, 3.6]**; (83.2%) 1.9 [1.8, 2.0]; (6.5%) 1.3 [1.1, 1.7]; (0.4%) 3.0 [2.9, 3.1]; (8.5%) — [—, —]; (1.3%)		3.8 [3.8, 3.9]; (76.6%) 3.5 [3.3, 3.6]; (10.1%) 2.1 [1.8, 2.5]; (0.6%) **4.4 [4.2, 4.5]**; (10.8%) — [—, —]; (1.9%)
Ethnicity^1a^					
Non-Spanish-Hispanic-Latino Spanish-Hispanic-Latino	**4.5 [4.5, 4.6]**; (92.4%) 2.4 [2.3, 2.5]; (7.6%)		**3.6 [3.5, 3.6]**; (91.7%) 2.2 [2.1, 2.3]; (8.3%)		**4.2 [4.1, 4.2]**; (90.8%) 2.6 [2.5, 2.7]; (9.2%)
Stage^1a,3a^					
In situ Localized Regional Distant Unknown/unstaged Not coded (Massachusetts)	0.0 [0.0, 0.0]; (0.5%) 0.4 [0.4, 0.4]; (10.0%) **2.8 [2.7, 2.8]**; (66.1%) 0.6 [0.6, 0.6]; (14.4%) 0.1 [0.1, 0.1]; (2.6%) 0.3 [0.3, 0.3]; (6.4%)		0.1 [0.1, 0.1]; (3.9%) **1.5 [1.5, 1.5]**; (42.3%) 1.1 [1.1, 1.1]; (31.5%) 0.4 [0.3, 0.4]; (10.4%) 0.2 [0.2, 0.2]; (5.5%) 0.2 [0.2, 0.2]; (6.5%)		0.1 [0.1, 0.2]; (3.7%) **1.4 [1.4, 1.4]**; (33.6%) 1.2 [1.2, 1.3]; (30.5%) 0.6 [0.6, 0.7]; (15.6%) 0.4 [0.4, 0.4]; (10.2%) 0.3 [0.2, 0.3]; (6.3%)
nSES (Yost Index)^2a,4a^					
Group 1 (lowest tertile) Group 2 Group 3 (highest tertile) Missing No match in Census data**	4.0 [3.9, 4.1]; (27.7%) 4.0 [3.9, 4.1]; (31.9%) 4.0 [3.9, 4.1]; (34.5%) 4.2 [3.8, 4.6]; (1.8%) — [—, —]; (4.1%)		3.1 [3.0, 3.2]; (28.2%) 3.2 [3.1, 3.2]; (32.2%) 3.2 [3.1, 3.3]; (34.0%) 3.0 [2.6, 3.4]; (1.6%) — [—, —]; (4.0%)		3.9 [3.8, 4.0]; (30.3%) 3.8 [3.7, 3.9]; (32.2%) 3.5 [3.4, 3.6]; (31.5%) 4.0 [3.6, 4.4]; (1.9%) — [—, —]; (4.2%)
RUCA^2a^					
All Urban Mostly Urban Mostly Rural All Rural No match in Census data**	**3.8 [3.7, 3.9]**; (61.1%) 4.4 [4.3, 4.5]; (20.9%) 4.6 [4.3, 4.8]; (7.4%) 4.4 [4.2, 4.6]; (6.5%) — [—, —]; (4.1%)		**3.1 [3.0, 3.1]**; (63.0%) 3.3 [3.2, 3.5]; (19.9%) 3.6 [3.3, 3.8]; [(7.0%) 3.3 [3.1, 3.5]; (6.1%) — [—, —]; (4.0%)		**3.6 [3.6, 3.7]**; (63.4%) 3.8 [3.7, 3.9]; (19.4%) 3.9 [3.7, 4.1]; (6.5%) 4.2 [4.0, 4.4]; (6.6%) — [—, —]; (4.2%)

*95% confidence interval; ** --- = statistic could not be calculated; **bolding** indicates majority groups that were significantly different, based on non-overlapping 95% confidence intervals within sociodemographic variables.

^1a^Incidence rates according to sex, race, and ethnicity reflect cases diagnosed in one of 21 SEER Program registries (data citation: Surveillance, Epidemiology, and End Results (SEER) Program (www.seer.cancer.gov) SEER*Stat Database: Incidence - SEER Research Plus Limited-Field Data, 21 Registries, Nov 2020 Sub (2000-2018) - Linked To County Attributes - Total U.S., 1969-2019 Counties, National Cancer Institute, DCCPS, Surveillance Research Program, released April 2021, based on the November 2020 submission).

^2a^Incidence rates according to the Yost Index and RUCA reflect cases diagnosed 2012-2016 in one of 18 SEER Program registries (data citation: Surveillance, Epidemiology, and End Results (SEER) Program (www.seer.cancer.gov) SEER*Stat Database: Incidence - SEER 18 Regs (Excl AK) Custom Data (with additional treatment fields), Nov 2018 Sub (2000-2016) <Vintage 2016 Pops by Tract 2000/2010 Mixed Geographies> - Linked To Census Tract Attributes - Time Dependent (2000-2016) - SEER 18 (excl AK) Census 2000/2010 Geographies with Index Field Quantiles, National Cancer Institute, DCCPS, Surveillance Research Program, released January 2020, based on the November 2018 submission). As a result, sample sizes were as follows: HPV-related OCPC n=21,493, non-HPV-related OCPC n=16,197, “other” OCPC n=18,785.

^3a^In situ cancers included only for stage-specific incidence rates. As a result, sample sizes were as follows: HPV-related OCPC n=26,282, non-HPV-related OCPC n=19,977, “other” OCPC n=21,184.

^4a^The Yost Index is a time-dependent composite score, constructed using a factor analysis from the following seven US Census variables: median household income, median house value, median rent, percent below 150% of poverty line, education Index, percent working class, and percent unemployed.

As an additional analysis, incidence rates were restricted to late-stage diagnoses (i.e., regional and distant stages) for nSES and RUCA. HPV-like OCPCs had the highest incidence rates for each nSES tertile. Incidence rates decreased with higher nSES for non-HPV-like and “other”-like OCPCs alike. Data is not shown as no differences were statistically significant. For RUCA late-stage analyses, incidence rates were largely similar to those including all stages (data not shown).

**Figure 3:**
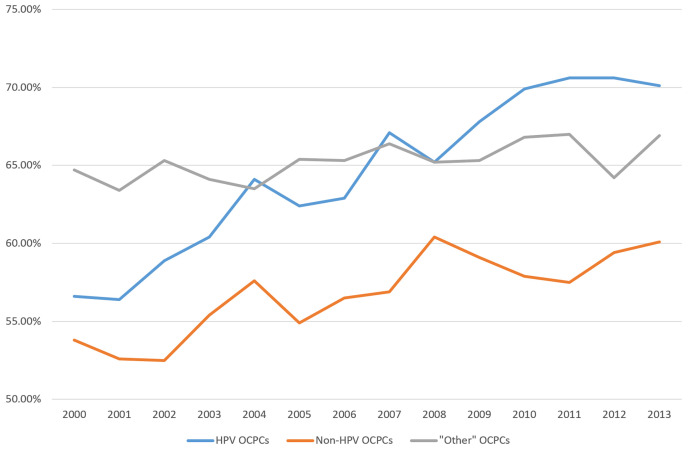
Recent trends in United States relative five-year survival rates of oral cavity and pharynx cancers according to groupings of human papillomavirus (HPV)-like association***. ***Surveillance, Epidemiology, and End Results (SEER) Program (www.seer.cancer.gov) SEER*Stat Database: Incidence - SEER Research Data, 18 Registries, Nov 2020 Sub (2000-2018) - Linked To County Attributes - Time Dependent (1990-2018) Income/Rurality, 1969-2019 Counties, National Cancer Institute, DCCPS, Surveillance Research Program, released April 2021, based on the November 2020 submission.

From 2000 to 2013, OCPC survival rates have varied by OCPC category (**Figure 3**). HPV-like OCPC survival rates have been increasing steadily. Increasing more slowly from 2003 to 2013, non-HPV-like OCPC survival rates have remained the lowest over time. “Other”-like OCPCs have remained steady with a survival rate hovering around 65% (**Figure 3**).

Survival rates also varied by sociodemographics across OCPC categories (**Table 2**). Survival rates were significantly higher among males for HPV-like OCPCs and females for both non-HPV-like and “other”-like OCPCs. Whites with HPV-like OCPCs had the highest survival rate; Asian or Pacific Islanders with non-HPV-like and “other”-like OCPCs faired minimally better than Whites. African Americans had a statistically significant lower survival rate among all HPV-like OCPC cases (**Table 2**). Survival rates were significantly higher among non-Hispanics for HPV-like and non-HPV-like OCPCs, but rates were similar among Hispanics and non-Hispanics for “other”-like OCPCs (**Table 2**). With advancing stage at diagnosis, survival rates fluctuated for HPV-like cancers and decreased for both non-HPV-like and “other”-like OCPCs. Regardless of OCPC categorization, distantly staged tumors at diagnosis had significantly lower survival rates compared to other stages in each group; *in situ* non-HPV-like OCPCs had a significantly higher survival rate than any other staged non-HPV-like OCPC (**Table 2**). Survival rates were significantly higher among residents of census tracts in the highest nSES tertile (and significantly lower in the lowest nSES tertile) for all OCPC categories, with HPV-like and non-HPV-like OCPCs having the highest and lowest rates, respectively. Although not significant, people in ‘mostly urban’ census tracts had the greatest survival rates among HPV-like and non-HPV-like OCPCs, while ‘all rural’ residents had the highest survival for those people with “other”-like OCPCs (**Table 2**).

**Table 2:** Relative five-year survival probabilities of oral cavity and pharynx cancer (OCPC) cases by demographic and census tract-based neighborhood socioeconomic status (nSES, Yost Index) and Rural-Urban Commuting Area (RUCA) according to groupings of human papillomavirus (HPV)-like association, for cases diagnosed from 2011 through 2017 with follow-up through 2018.

	HPV-like OCPCs 5-year Survival, %, [95% CI]*	Non-HPV-like OCPCs 5-year Survival, %, [95% CI]*	“Other”-like OCPCs 5-year Survival, %, [95% CI]*
Sex^1b^			
Male Female	**72.2 [71.3, 73.0]** 64.9 [63.0, 66.8]	58.2 [56.9, 59.5] **62.7 [61.1, 64.3]**	63.2 [62.1, 64.3] **75.2 [73.8, 76.7]**
Race^1b^			
White African American American Indian/Alaska Native Asian or Pacific Islander Unknown	72.9 [72.1, 73.7] **50.5 [47.7, 53.2]** 67.0 [56.7, 75.4] 69.5 [64.7, 73.8] 97.0 [90.7, 99.1]	60.7 [59.5, 61.8] 43.2 [39.7, 46.6] 48.8 [35.4, 60.9] 64.5 [61.1, 67.6] 95.3 [88.3, 98.2]	67.8 [66.8, 68.8] 55.7 [52.8, 58.4] 60.0 [49.6, 69.0] 69.4 [67.0, 71.6] 97.5 [93.6, 99.1]
Ethnicity^1b^			
Non-Hispanic Hispanic	**71.4 [70.6, 72.2]** 65.9 [63.0, 68.7]	**60.5 [59.5, 61.6]** 53.9 [50.3, 57.3]	67.0 [66.1, 68.0] 68.7 [66.0, 71.2]
Stage^1b,3b^			
In situ Localized Regional Distant Unknown/unstaged	71.8 [58.8, 81.4] 80.3 [77.7, 82.6] 76.6 [75.7, 77.4] **47.9 [46.1, 49.6]** 59.8 [54.1, 64.9]	**94.4 [88.8, 97.3]** 80.1 [78.6, 81.5] 47.8 [46.1, 49.5] **28.6 [26.3, 30.9]** 52.3 [47.2, 57.1]	98.6 [91.7, 98.6] 91.4 [90.1, 92.5] 62.3 [60.6, 63.9] **38.0 [36.3, 39.7]** 55.6 [52.1, 59.0]
nSES (Yost Index)^2b,4b^			
Group 1 (lowest tertile) Group 2 Group 3 (highest tertile) Missing No match in Census data	**56.5 [54.9, 58.0]** 70.2 [68.8, 71.6] **80.4 [79.2, 81.6]** 74.1 [65.9, 80.6] 64.5 [60.5, 68.2]	**48.4 [46.5, 50.3]** 59.0 [57.2, 60.8] **66.5 [64.6, 68.2]** 54.5 [45.1, 63.0] 59.7 [54.6, 64.5]	**57.6 [56.0, 59.2]** 66.6 [65.0, 68.1] **73.5 [72.0, 75.0]** 65.5 [54.5, 74.4] 65.2 [60.6, 69.3]
RUCA^2b^			
All Urban Mostly Urban Mostly Rural All Rural No match in Census data	68.8 [67.8, 69.8] 73.2 [71.5, 74.8] 70.3 [67.3, 73.1] 68.8 [65.5, 71.7] 64.5 [60.5, 68.2]	56.6 [55.3, 57.9] 63.3 [61.0, 65.5] 59.6 [55.4, 63.5] 56.0 [51.6, 60.1] 59.7 [54.6, 64.5]	65.7 [64.6, 66.8] 66.3 [64.1, 68.3] 63.2 [59.3, 66.9] 70.2 [66.3, 73.8] 65.2 [60.6, 69.3]

*95% confidence interval; **bolding** indicates groups with a highest or lowest survival rate among ‘known’ levels that were significantly different, based on non-overlapping 95% confidence intervals within sociodemographic variables.

^1b^Surveillance, Epidemiology, and End Results (SEER) Program (www.seer.cancer.gov) SEER*Stat Database: Incidence - SEER Research Data, 18 Registries, Nov 2020 Sub (2000-2018) - Linked To County Attributes - Time Dependent (1990-2018) Income/Rurality, 1969-2019 Counties, National Cancer Institute, DCCPS, Surveillance Research Program, released April 2021, based on the November 2020 submission.

^2b^Survival rates according to the Yost Index and RUCA reflect cases diagnosed 2009-2011 and followed into 2016 in one of 18 registries, Surveillance, Epidemiology, and End Results (SEER). Program (www.seer.cancer.gov) SEER*Stat Database: Incidence - SEER 18 Regs (Excl AK) Custom Data (with additional treatment fields), Nov 2018 Sub (2000-2016) <Vintage 2016 Pops by Tract 2000/2010 Mixed Geographies> - Linked To Census Tract Attributes - Time Dependent (2000-2016) - SEER 18 (excl AK) Census 2000/2010 Geographies with Index Field Quantiles, National Cancer Institute, DCCPS, Surveillance Research Program, released January 2020, based on the November 2018 submission.

^3b^In situ cancers included only for stage-specific incidence rates.

^4b^The Yost Index is a time-dependent composite score, constructed using a factor analysis from the following seven US Census variables: median household income, median house value, median rent, percent below 150% of poverty line, education Index, percent working class, and percent unemployed.

As an additional analysis, survival rates were also restricted to late-stage diagnoses (i.e., regional and distant stages) for nSES and RUCA. Those residing in census tracts in the highest nSES tertile for each of the three OCPC categories maintained a significantly better survival rate than any other tertile within each OCPC category (except for non-HPV-like which was marginally significant). Comparing the survival rates of the highest nSES tertile across OCPC categories, HPV-like OCPCs had the highest rate, followed by “other”-like OCPCs, and then non-HPV-like OCPCs (**Supplementary Figure 1**). Similarly, the lowest nSES tertile for each OCPC category had a significantly worse survival rate than any other tertile in the group; those residing in the lowest tertile and having a non-HPV-like OCPC had the lowest survival rate of all (**Supplementary Figure 1**). For RUCA late-stage analyses, survival rates were largely similar to those when all diagnosis stages were included initially (data not shown).

## Discussion

These findings suggested that incidence and survival rates varied by OCPC category and by demographics. HPV-like OCPC incidence and survival rates were distinct from non-HPV-like OCPC rates for categories of age, sex, race, ethnicity, stage of diagnosis, nSES, and RUCA. “Other”-like and non-HPV-like OCPCs were more similar, sharing higher incidence rates for categories within sex, race, stage at diagnosis, and nSES (late-stage diagnoses only) variables while better survival rates were shared among the same categories of sex and stage. Uniquely, “other”-like OCPC incidence rates decreased as nSES increased; patterns were not seen for HPV-like and non-HPV-like OCPCs. Across all OCPCs, survival was significantly higher for those in ‘all rural’ census tracts.

Many of these HPV-related and non-HPV-related OCPC findings were supported by previous research. Prior studies found that HPV-related OCPCs were more common among younger ages (14, 38, 39), males (14, 20, 22, 39, 40), non-Hispanics (38, 41), and Whites (14, 20, 22, 27, 38–40, 42) than non-HPV-related OCPC groups. Compared to non-HPV-related OCPCs, HPV-related OCPCs also tended to be diagnosed at more advanced stages (27, 39). Survival rates have been shown to be higher for HPV-related than for non-HPV-related OCPCs (14, 27, 42). Consistent with these findings, non-Whites with HPV-related (27, 42) and non-HPV-related (26, 27, 42) OCPCs, alike, had lower survival rates compared to Whites.

The current study found that OCPC incidence rates did not vary by nSES for HPV-like OCPCs; incidence rates decreased with increasing nSES for late-stage non-HPV-like OCPCs. Prior research reported that males with HPV-positive head and neck squamous cell carcinomas were more likely to live in zip codes with higher median household incomes and educational attainments than males with HPV-negative cancers (38). Another study found higher odds of HPV-related squamous cell carcinoma metastasis among census tracts with the fewest high school graduates (40). The present findings of increasing survival rates with increasing tertile of nSES across OCPC categories are supported by other studies (26, 27).

The current findings concerning higher incidence rates of HPV-like OCPCs in non-urban census tracts are supported by county-based incidence rates reported by others for oropharyngeal cancer (20, 28). Further, oropharyngeal cancers in another study were significantly more likely to be HPV-positive among rural county residents (38). For each of the OCPC categories, the present survival findings did not vary considerably for RUCA categories. One study reported a marginally significant survival benefit among rural HPV-negative cases, but not for HPV-positive cases (26).

This study was strengthened by SEER Program data, which includes many US OCPC cases, representing complete and quality data from a large racially and ethnically heterogeneous population (43). Further, residences of SEER cases are linked by census tract to multiple domains of nSES measures, including core SES dimensions of income, education, and employment. The Yost Index, used to assess nSES, has face validity, is transparent and independently reproducible (44), and, compared to counties, census tracts more closely resemble neighborhoods, allowing for a more accurate nSES representation (45). Additionally, OCPCs which were not exclusively identified as HPV-like nor non-HPV-like OCPCs (i.e., “other”-like OCPCs) were also retained, maximizing the number of OCPCs investigated.

This study also had limitations. A primary limitation in these analyses pertains to the potential for misclassification in the categorization of HPV-like and non-HPV-like OCPCs as HPV status was unknown in these SEER datasets and there are no universally accepted HPV status specific categories. Supported by national-level (US) evidence of HPV-tested OCPC sub-anatomies (13), an accepted methodology of categorizing OCPC specific anatomies and histologies based on primary risk factors (14) was replicated herein to minimize any biased findings. Another limitation is the lack of comorbidity and patient behavior data, including OCPC screening behavior, in SEER; these factors may be associated with survival. Further, nSES and RUCA categories were assessed cross-sectionally; neighborhoods may have changed over time. In addition, nSES and RUCA categories may not represent individual SES and rurality/urbanity. Another limitation was that different, yet overlapping, datasets were required to examine incidence and survival rates and that different sets of years (2014-2018 and 2012-2016) were available to examine incidence rates according to levels of individual demographic factors and census tract-based measures, respectively.

Especially when OCPC HPV status is unavailable as in this investigation, every effort to coalesce varying OCPC terminology and related categories into one coherent list is vital for ensuring that OCPC incidence rates do not go unchecked and that higher risk groups do not go unrecognized. This is especially concerning given that the current study found incidence and survival rates of OCPCs differed significantly by aetiological risk factor category and reported considerable variation over time and across sociodemographic characteristics, including nSES and RUCA categories. Medical providers and public health personnel can incorporate the current study’s findings into their ongoing efforts to minimize the OCPC burden by tailoring and targeting, respectively, their OCPC education approaches to high risk groups. For example, public health can create targeted posters and brochures aimed at males, Whites, non-Hispanics, and rural residents to make them aware of their greater OCPC vulnerability. Additionally, they should make concerted efforts to inform females, minority races and ethnicities, and poorer populations of their potentially lower likelihood of survival should they be diagnosed with an OCPC, emphasizing the importance of routine screening in these groups to detect their cancers earlier. Similarly, medical providers can utilize these risk factors to individually assess their patients’ risk so they may further tailor their patient education efforts and intervene appropriately. This study’s consideration of “other” HPV-like OCPCs gives health-related professionals a jumpstart at beginning to understand and inform higher risk groups of the lesser-known cancers, potentially preventing them from ever becoming an area of public health concern. Ideally, public health and medicine will work in unison to educate any OCPC high risk populations with clear and consistent messaging of the aforementioned OCPC incidence- and survival-based risk factors to make the greatest impact on OCPC rates. The recent availability of a SEER dataset containing HPV status for a subset of head and neck cancers may help further clarify which specific anatomies and histologies should be classified into the categories the current study estimated herein. A better understanding of the epidemiological composition of these multiple OCPC aetiological risk factor-based categories will allow physicians and public health professionals to continue to develop more appropriately and effectively tailored and targeted practices to minimize the OCPC burden for individuals and communities alike.

## Data availability statement

Publicly available datasets were analyzed in this study. This data can be found here: www.seer.cancer.gov.

## Ethics statement

Ethical review and approval was not required for the study on human participants in accordance with the local legislation and institutional requirements. Written informed consent for participation was not required for this study in accordance with the national legislation and the institutional requirements.

## Author contributions

All authors approved the final manuscript version and are accountable for all research-related activities herein. KJ: conceptualization, formal analysis, visualization, writing-original draft, writing-review & editing, project administration. JF: data curation, formal analysis, validation, visualization, writing-original draft, writing-review & editing. EP: conceptualization, resources, writing-review & editing, supervision.

## Funding

KJ (P01 CA229143-S1) has National Cancer Institute (NCI) funding through the National Institutes of Health (NIH).

## Conflict of interest

EP receives grant funding through the university from Pfizer and Merck Foundation.

The remaining authors declare that the research was conducted in the absence of any commercial or financial relationships that could be construed as a potential conflict of interest.

## Publisher’s note

All claims expressed in this article are solely those of the authors and do not necessarily represent those of their affiliated organizations, or those of the publisher, the editors and the reviewers. Any product that may be evaluated in this article, or claim that may be made by its manufacturer, is not guaranteed or endorsed by the publisher.

## Supplementary material

Supplementary Figure 1 Relative five-year survival rates of late-stage oral cavity and pharynx cancers by census tract-based neighborhood socioeconomic status (nSES, Yost Index) according to groupings of human papillomavirus (HPV)-like association ^^^. ^^^Surveillance, Epidemiology, and End Results (SEER) Program (www.seer.cancer.gov) SEER*Stat Database: Incidence - SEER 18 Regs (Excl AK) Custom Data (with additional treatment fields), Nov 2018 Sub (2000-2016) <Vintage 2016 Pops by Tract 2000/2010 Mixed Geographies> - Linked To Census Tract Attributes - Time Dependent (2000-2016) - SEER 18 (excl AK) Census 2000/2010 Geographies with Index Field Quantiles, DCCPS, Surveillance Research Program, released January 2020, based on the November 2018 submission. Survival rates reflecting missing or unmatchable census tracts (for the Yost Index) are incalculable (based on inestimable denominators) or not shown. The Yost Index is a time-dependent composite score, constructed using a factor analysis from the following seven US Census variables: median household income, median house value, median rent, percent below 150% of poverty line, education Index, percent working class, and percent unemployed.
