# Supplementary figures and images for: Distinct sociodemographic differences in incidence and survival rates for human papillomavirus (HPV)-like, non-HPV-like, and “other”-like oral cavity and pharynx cancers: An analysis of Surveillance, Epidemiology and End Results (SEER) Program data
Source: Front Oncol. 2022 Aug 18;12:980900. doi: 10.3389/fonc.2022.980900 (PMC9444004; doi:10.3389/fonc.2022.980900)

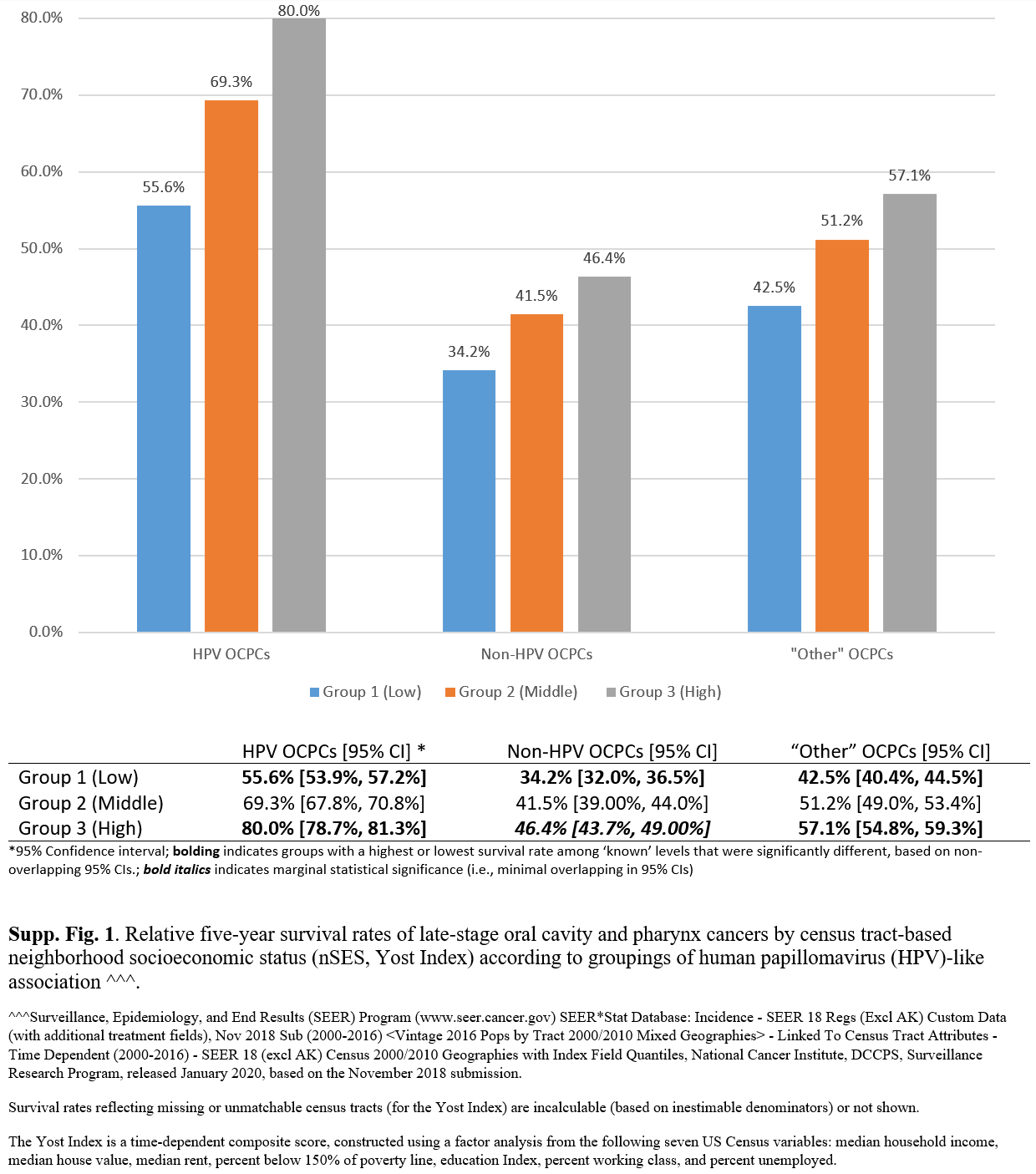

Supplement: Supplementary Figure 1 — Relative five-year survival rates of late-stage oral cavity and pharynx cancers by census tract-based neighborhood socioeconomic status (nSES, Yost Index) according to groupings of human papillomavirus (HPV)-like association ^^^. ^^^Surveillance, Epidemiology, and End Results (SEER) Program (www.seer.cancer.gov) SEER*Stat Database: Incidence - SEER 18 Regs (Excl AK) Custom Data (with additional treatment fields), Nov 2018 Sub (2000-2016) - Linked To Census Tract Attributes - Time Dependent (2000-2016) - SEER 18 (excl AK) Census 2000/2010 Geographies with Index Field Quantiles, DCCPS, Surveillance Research Program, released January 2020, based on the November 2018 submission. Survival rates reflecting missing or unmatchable census tracts (for the Yost Index) are incalculable (based on inestimable denominators) or not shown. The Yost Index is a time-dependent composite score, constructed using a factor analysis from the following seven US Census variables: median household income, median house value, median rent, percent below 150% of poverty line, education Index, percent working class, and percent unemployed. [file Image_1.tif]
